# Geological Survey

**Published:** 1847-06

**Authors:** 


ARTICLE VII.

### GEOLOGICAL SURVEY.

The Corps of Geologists recently appointed by the General Government to survey the mineral lands authorized by Congress to be brought into the market, is made up of some of the most scientific men in the country.

David Dale Owen, of Indiana, formerly geologist of the State, distinguished for his ability and correctness in this department of science is chief; with Dr. J. G. Norwood, of Indiana, Col. Charles Whittelsey, of Ohio, Prof. Randall, of Cincinnati, B. C. Macy, of New York, and Dr. Shumard, of Kentucky, as assistants.

The service is expected to continue for several years, and we will look forward with much interest to the reports of their progress, which we suppose will be sent in from time to time.

We understand the principal field of their operations will be the northern part of Wisconsin and the new territory of Minesota.

